# Single-Cell Atlas of Atherosclerosis Patients by Cytof: Circulatory and Local Immune Disorders

**DOI:** 10.14336/AD.2023.0426-1

**Published:** 2024-02-01

**Authors:** Xiaolong Ya, Hao Li, Peicong Ge, Yiqiao Xu, Zechen Liu, Zhiyao Zheng, Siqi Mou, Chenglong Liu, Yan Zhang, Rong Wang, Qian Zhang, Xun Ye, Wenjing Wang, Dong Zhang, Jizong Zhao

**Affiliations:** ^1^Department of Neurosurgery, Beijing Tiantan Hospital, Capital Medical University, Beijing, China.; ^2^Beijing Institute of Hepatology, Beijing YouAn Hospital, Capital Medical University, Beijing, China.; ^3^China National Clinical Research Center for Neurological Diseases, Beijing, China.; ^4^Capital Medical University, Beijing, China.; ^5^Department of Biostatistics, Harvard School of Public Health, Boston, USA.; ^6^Peking Union Medical College Hospital, Chinese Academy of Medical Sciences and Peking Union Medical College, Beijing, China.; ^7^University of Chinese Academy of Sciences, Beijing, China.

**Keywords:** Atherosclerosis, CyTOF, RNA-seq analysis, immune environment

## Abstract

Atherosclerosis (AS) is a common underlying pathology of coronary artery disease, peripheral artery disease, and stroke. The characteristics of immune cells within plaques and their functional relationships with blood are crucial in AS. In this study, Mass cytometry (CyTOF), RNA-sequencing and immunofluorescence were combined to comprehensively analyze plaque tissues and peripheral blood from 25 AS patients (22 for Mass cytometry and 3 for RNA-sequencing), as well as blood from 20 healthy individuals. The study identified a complexity of leukocytes in the plaque, including both defined anti-inflammatory and pro-inflammatory subsets such as M2-like CD163+ macrophages, Natural killer T cells (NKT), CD11b+ CD4+ T effector memory cells (Tem), and CD8+ terminally differentiated effector memory cells (TEMRA). Functionally activated cell subsets were also found in peripheral blood in AS patients, highlighting the vivid interactions between leukocytes in plaque and blood. The study provides an atlas of the immune landscape in atherosclerotic patients, where pro-inflammatory activation was found to be a major feature of peripheral blood. The study identified NKT, CD11b+ CD4+ Tem, CD8+ TEMRA and CD163+ macrophages as key players in the local immune environment.

## INTRODUCTION

Atherosclerosis plaques are the most common underlying pathology of coronary artery disease (CAD), peripheral artery disease (PAD), and stroke [1-3]. The occurrence and development of plaques are influenced by various external environmental factors, which act through changes in the immune microenvironment [4, 5]. Previous studies have found that systemic immune alterations such as T cell activation, B cell regulation, and cytokine production have been implicated in atherosclerosis [6, 7]. At the same time, local immune responses, including the infiltration of inflammatory cells such as macrophages and T cells, have been shown to be key drivers of plaque progression [8, 9]. Despite the considerable advances made in our understanding of the immune mechanisms underlying atherosclerosis, the identification of key therapeutic targets that modulate immune activation and reduce plaque inflammation without compromising systemic immunity still is an ongoing challenge.

Recently, single-cell analyses have been used to study murine and human atherosclerosis, highlighting their potential to systematically reveal immunological diversity and decipher how immune cell alterations contribute to human disease [10-13]. Here, we combined Mass cytometry (CyTOF), a single-cell technology, with RNA-sequencing and immunofluorescence to precisely define leukocytes in human atherosclerosis plaques and the blood. Our results indicated a complexity of leukocytes in the plaque with both defined anti-inflammatory and pro-inflammatory leukocyte subsets including the increasing M2-like macrophages, activated NKT cells and activated memory CD8+ T cells. In addition, we found a variety of functionally activated cell subsets in peripheral blood in AS patients and vividly interactions between circulating leukocytes between plaque and blood. This study provides a comprehensive atlas of the immune landscape in atherosclerotic patients, shedding light on the pro-inflammatory activation in peripheral blood and identifying key immune cell subsets that contribute to the local immune environment of atherosclerotic plaques, which may help develop novel immunotherapeutic strategies for atherosclerosis.

## MATERIALS AND METHODS

### Human Specimens and Ethics Statements

This research was approved by the Institutional Review Board (IRB) and Ethics Committee of Beijing Tiantan Hospital (Beijing, China), and all patients and volunteers provided written informed consent.

Between August and November 2021, 25 atherosclerotic patients who underwent carotid atherectomy at Beijing Tiantan Hospital were enrolled in this study (Inclusion criteria: A. patients with confirmed carotid artery stenosis through DSA, CTA, MRA, or ultrasound; B. patients undergoing carotid atherectomy with preoperative peripheral blood and plaques available; C. age above 50 years old. Exclusion criteria: A. patients with tumor diseases; B. patients with infectious diseases; C. patients with severe liver/renal damages; D. patients who received chemotherapy, radiotherapy, or any medication that might impair the systemic immune system). Blood and plaques were collected from each patient. The specimens from 22 atherosclerotic patients were used for CyTOF analysis, and the specimens from another 3 patients were analyzed for RNA-seq analysis. Additionally, peripheral blood was collected from 20 healthy volunteers who met the inclusion and exclusion criteria (Inclusion criteria: A. without carotid artery stenosis confirmed by DSA, CTA, MRA or ultrasound; B. age>50 years. Exclusion criteria: A. with tumor diseases; B. with infectious diseases; C. with severe liver/renal damages; D. with received chemotherapy, radiotherapy, or any medications that might impair the systemic immune system). Detailed clinical information of 22 patients and 20 volunteers can be found in Supplementary Table 1.

### Plaque Tissue Single-Cell Dissociation

Atherosclerotic plaque tissues were washed with Dulbecco's phosphate-buffered saline (DPBS, Sigma-Aldrich, United States) within 1 hour after surgery. Each specimen was digested at 37°C for 1 hour using miscible liquids that contained collagenase type IV (GIBCO, Gaithersburg, United States), DNase (Sigma, DN25), hyaluronidase (Sigma, H3506), collagenase type XI (Sigma, C7657), and collagenase type II (Sigma, C6885). The mixture was then filtered through a 70 μm cell strainer with DPBS and washed with red blood cell (RBC) lysis buffer (BD Biosciences, United States). The dissociated cell suspension was washed once with DPBS and resuspended in 1 mL of staining buffer (DPBS containing 5% fetal bovine serum, ScienCell, United States).

### Blood Single-Cell Dissociation

Upon admission, fresh blood samples were collected in EDTA anticoagulation tubes and then transferred to SepMate PBMC isolation tubes containing Ficoll (STEMCELL Technologies, Canada). After centrifugation (10 min at 1200 g, minimal braking), the cells were washed with RBC lysis buffer and subsequently washed twice with staining buffer.

### Mass Cytometry

At admission, fresh blood samples were collected in EDTA anticoagulation tubes. Subsequently, the samples were transferred into SepMate PBMC isolation tubes containing Ficoll (STEMCELL Technologies, Canada) and centrifuged (10 min at 1200 g, minimal braking). The cells were washed with RBC lysis buffer and then twice with staining buffer. To distinguish a broad range of immune cells, we designed a panel of 39 antibodies purchased in a purified form from Biolegend (San Diego, United States) and conjugated in-house using the Maxpar X8 Multimetal Labeling Kit (Fluidigm, United States) following the manufacturer's instructions. The list of antibodies and reporter isotopes used is provided in Supplementary Table 2. Following the addition of cisplatin-195Pt (Fluidigm, 201064) as a viability dye, cell samples were washed and stained with cell surface antibodies on ice for 30 min. Subsequently, the samples were permeabilized at 4°C overnight and stained with intracellular antibodies for 30 min on ice. After washing, the antibody-labeled samples were incubated in 0.125 nM intercalator-Ir (Fluidigm, United States) diluted in phosphate-buffered saline (PBS, Sigma-Aldrich, United States) containing 2% formaldehyde and stored at 4°C until mass cytometry examination. Before acquisition, the samples were washed with deionized water and re-suspended at a concentration of 1 x 106 cells/mL in deionized water containing a 1:20 dilution of EQ Four Element Beads (Fluidigm, United States). The samples were then examined using CyTOF2 mass cytometry (Fluidigm, United States)

### CyTOF Data Analysis

CyTOF data were acquired in the form of .fcs files using the CyTOF2 system. The addition of EQ Four Element Beads allowed us to use a MATLAB-based normalization technique. The data were uploaded to Cytobank (https://premium.cytobank.cn). First, the beads were filtered, and active cells were selected based on a specific gate. Then, CD45+ cells were gated to explore the leukocyte subsets (see Supplementary Figure 1 for details). Further analysis was performed using the automated dimensionality reduction algorithm, FlowSom, implemented in R. The results were visualized using viSNE, a visual dimensionality reduction algorithm.

### RNA Extraction and Library Construction

RNA sequencing was performed on peripheral blood and plaque tissues from three patients. In addition, peripheral blood samples from four healthy individuals were used as blank controls. After the peripheral blood samples were centrifuged (10 minutes at 1200 g, with minimal braking), the white blood cell layer was extracted and washed with DPBS (Sigma-Aldrich, United States). The plaque tissues were washed with DPBS (Sigma-Aldrich, United States) within one hour after surgical resection, and the TRIzol method was also used to extract total RNA. Sequencing libraries were generated using rRNA-depleted RNA with an NEBNext Ultra Directional RNA Library Prep Kit for Illumina (NEB, MA) following the manufacturer's recommendations. We then performed paired-end sequencing on an Illumina NovaSeq 6000 (Illumina, USA) as recommended by the supplier. After cluster generation, the libraries were sequenced on the Illumina HiSeq platform, generating 150-bp paired-end reads.

### Quality Control and Data Analysis

To obtain high-quality data, we first processed the raw fastq files through in-house Perl scripts. We downloaded the reference genome and gene model annotation files directly from the genome website and built the index of the reference genome using Bowtie2 v2.2.8. Then, we aligned the paired-end clean reads to the reference genome using Hisat2 v2.0.5. We downloaded the Hg19 RefSeq (RNA sequences, GRCh37) from the UCSC Genome Browser (http://genome.ucsc.edu) and aligned the clean reads with both genome hg19 and transcript reference using STAR v2.2.1. The gene expression was calculated by RSEM v1.3.0 using FPKM (fragments per kilobase of exon per million fragments mapped). We compared RNA-seq data from peripheral blood between healthy individuals and atherosclerotic patients and considered FDR < 0.05 as statistically significant. We used R for gene expression data analysis. The data presented in the study are deposited in the National Genomics Data Center repository, accession number OMIX002548.

### Histology and Immunofluorescence Staining

Plaques from five patients were fixed overnight in 4% formalin (4°C) and embedded in paraffin blocks for paraffin sections. Hematoxylin-eosin staining was performed according to routine experimental procedures. Hematoxylin and eosin (H&E) staining was performed as previously described (see Supplementary Figure 2 for details). Multicolor immunofluorescence was performed following a modified protocol mentioned in reference [14, 15]. Briefly, paraffin sections (4 um) were deparaffinized by baking at 70°C for at least 1 hour. Slides were then immersed in fresh xylene (X5-4, ThermoFisher) for 30 min and rehydrated in descending concentrations of ethanol (412811, Gold Shield) before being loaded into slide chambers containing 1X Target Retrieval Solution, pH9 (S236784-2, Agilent). Heat-induced antigen retrieval (HIER) was then performed using a PT Link Pre-Treatment Module (Dako, Agilent) at 97°C for 10 min. After antigen retrieval, slide chambers were removed from the module and allowed to equilibrate to room temperature for 30 min. Tissue sections were then encircled on slides using a polyacrylamide gel pen (Bondic). The slides were washed twice with 1X TBS IHC wash buffer containing Tween20 (935B-09, Cell Marque) at room temperature for 5 minutes. Slides were blocked for 1 hour at room temperature using bovine serum albumin (BSA) to prevent nonspecific antibody binding. Sections were sequentially stained for four different antigens, including anti-CD45 (Abcam, ab40763), anti-CD68 (Abcam, ab213363), anti-CD163 (Abcam, ab182422), and anti-HLA_DR (Abcam, ab92511) and anti-CD161 (Abcam, ab302564). Isotype negative controls were performed with an isotype-matched antibody, rabbit IgG (Abcam, ab125912), used as the negative control for all antibodies. Each staining step involved blocking with bovine serum albumin, application of primary antibody and corresponding secondary horseradish peroxidase-conjugated polymer antibodies, followed by the covalent binding of a different fluorophore using tyramide signal amplification (NEL703001KT, NEL741001KT, NEL744001KT) (AKOYA Biosciences). Fluorescent images were acquired on a Zeiss LSM880 NLO microscope, and H&E images were obtained using Zeiss Axio Scope Al. The experiment was repeated three times for each sample, and the data were analyzed by two independent observers in a blinded fashion.

### Statistical analysis

The clinical variables of the CyTOF cohort were presented as either mean ± standard deviation (SD) for continuous variables or as the number (n) and percentage (%) for categorical variables. The statistical analysis of categorical variables was performed using the chi-squared test, while the one-way ANOVA test was used for the statistical analysis of continuous variables. Boxplots were used to compare cell frequencies and beanplots were used to compare marker expressions between two groups. The median ± inter-quartile range (IQR) was shown on the plots. The Wilcoxon Rank Sum Test was utilized to detect differences among them. SPADEVizR method was used to analyze the differences in cell composition [16]. A p-value less than 0.05 was considered statistically significant. All statistical analyses were conducted using R software.

**Figure 1.**
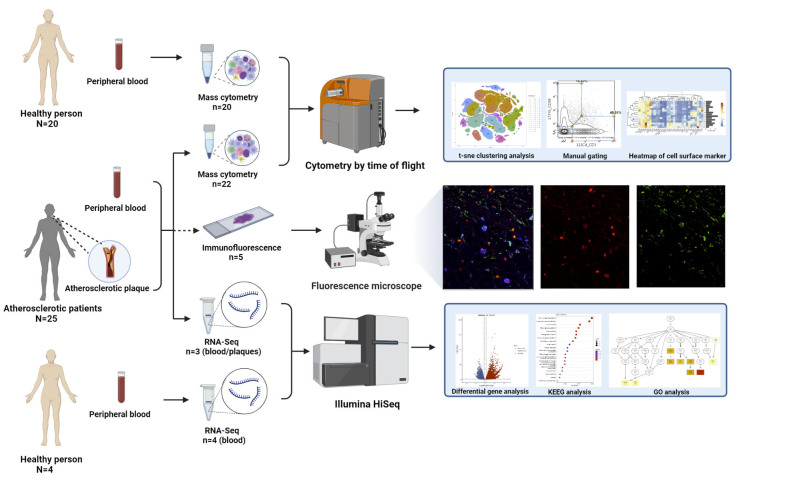
**Workflow**. The study included peripheral blood and plaque tissues obtained from 25 patients and peripheral blood from 20 healthy individuals. Mass cytometry was performed on 22 plaques and the findings were confirmed using immunofluorescence on paraffin sections of 5 remnants that were paraffin-embedded. In addition, atherosclerotic plaques and paired blood from 3 more patients, as well as peripheral blood from 4 healthy volunteers, were analyzed for RNA-seq analysis. The workflow of the study is depicted in this figure (created using Biorender).

## RESULTS

### Single-cell immunophenotyping of samples.

**Figure 2.**
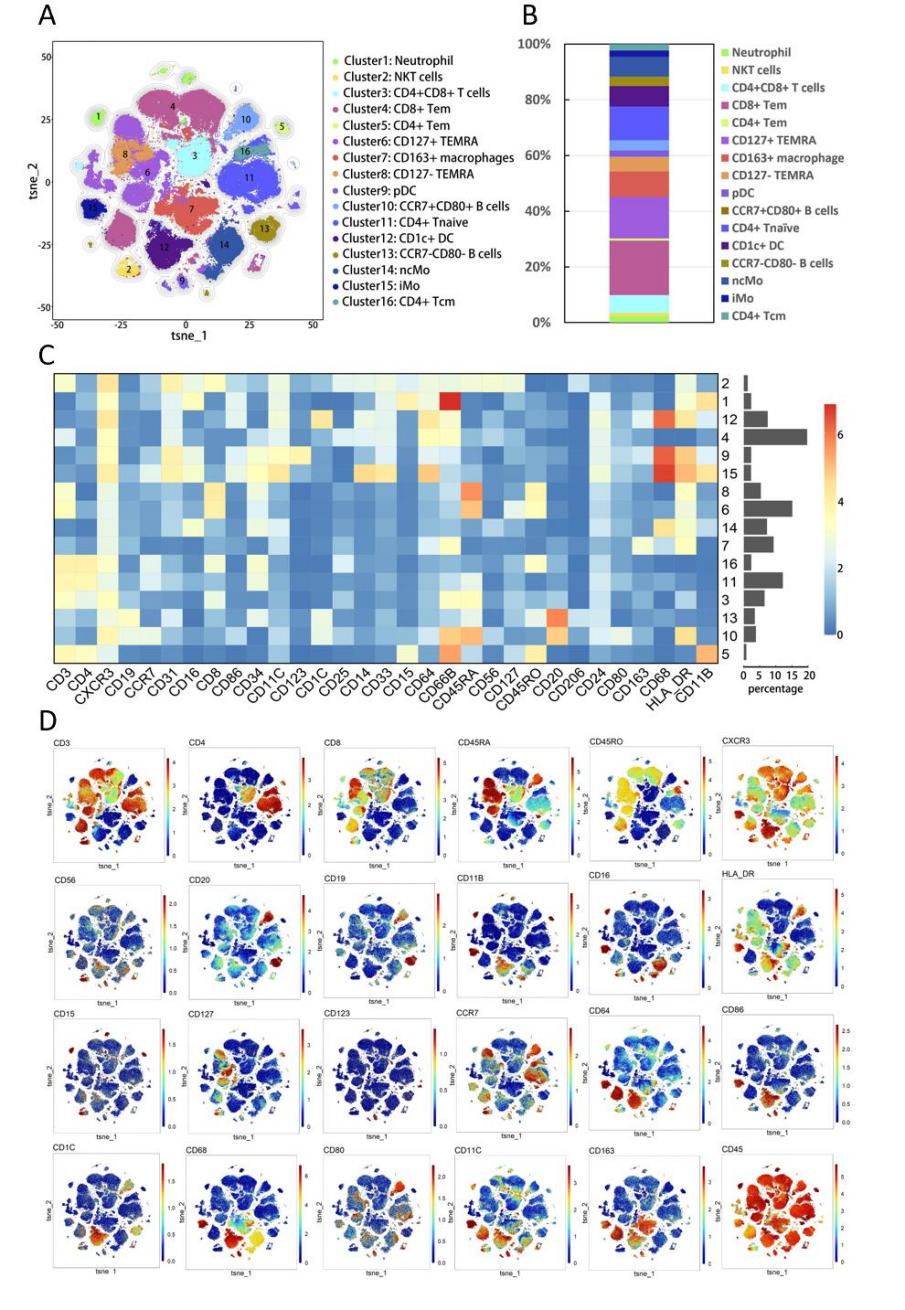
**Cell subsets in all samples (including peripheral blood from 20 healthy individuals, plaque tissues and paired blood from 22 patients)**. (**A**) Clustering analysis using t-SNE dimension reduction method identified 16 distinct cell subsets, which are visualized in the plot. (**B**) The proportions of each cluster in different samples are displayed in a stacking histogram. (**C**) A heatmap shows the relative expression levels of selected markers in the 16 t-SNE clusters. (**D**) The spectral colors overlaid on the t-SNE plot illustrate the marker expression patterns of the 16 cell subsets.

To provide a comprehensive analysis of the immune cell subsets in the samples, Unsupervised Flowsom cluster analysis was performed on samples from 22 atherosclerotic patients (including peripheral blood and paired plaques) and 20 healthy donors (peripheral blood) simultaneously (Fig. 1). The clinical characteristics of the enrolled individuals are summarized in Supplementary Table 1. A total of 16 subsets with distinct phenotypes were identified using specific surface markers to distinguish specific immune cell types (Figure 2A). Lymphoid cells accounted for 68.0% of the identified subsets, while mononuclear macrophages accounted for 29.7%. The most abundant immune cell subset was CD8+T cells (cluster 4) which accounted for 19.5%, whereas CD4+T cells (cluster 5) were the least abundant, accounting for only 0.8% (Fig. 2B). Finally, all samples were mapped using viSNE, and surface marker expression was visualized using heatmaps and spectrum maps (Fig. 2C and D).

**Figure 3.**
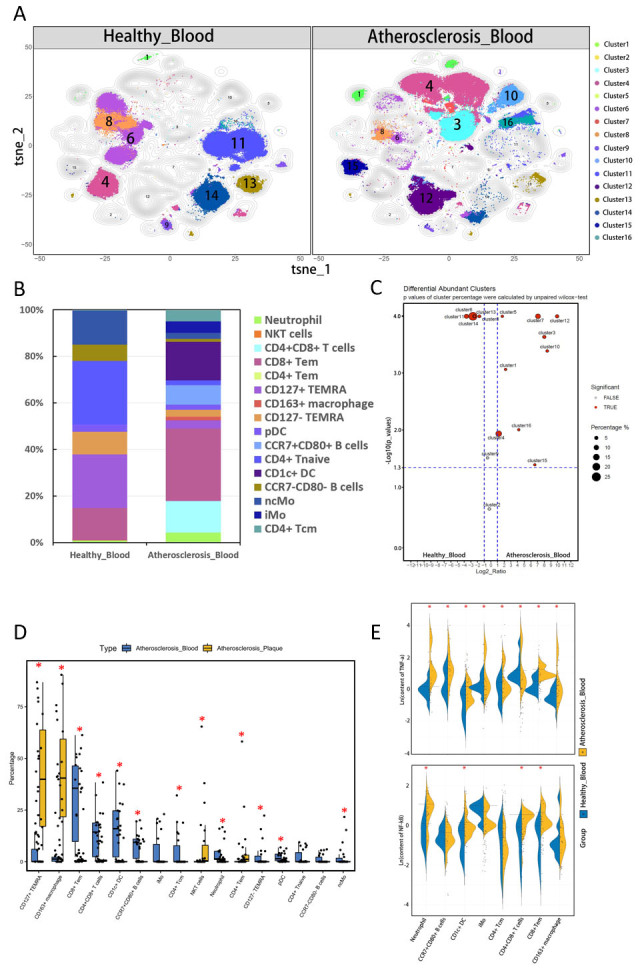
**Comparison of PBMC between atherosclerotic patients (n=22) and healthy controls (n=20)**. (**A**) The t-SNE dimension reduction method was used to display the components of PBMC in controls and patients. (**B**) Stacked histograms illustrate the composition and percentage of PBMC in 22 patients and 20 controls. (**C**) SPADEVizR method was utilized to analyze the differences in cell composition, which are displayed on the volcanic map. (**D**) Boxplots display the differences in the percentage of each subset between healthy controls and atherosclerotic patients. Asterisks indicate subsets with statistically significant differences (P < 0.05). (**E**) Functional analysis was conducted to compare the levels of NF-kB and TNF-α expressed by the same clusters enriched in patients (identified in C) with the counterpart in healthy controls. Asterisks indicate groups with statistically significant differences (P < 0.05).

### Circulating immune environment in atherosclerosis was constructed by a variety of proinflammatory cell subtypes

To investigate the differences in the composition of peripheral blood mononuclear cells (PBMC), we compared the immune subsets between controls and patients. T cells were the most abundant immune cell type in both controls and patients. However, healthy donors were primarily composed of CD45RA+CD45RO- naïve T cells (cluster11, 27.3%), while patients were dominated by CD45RA-CD45RO+ memory T cells (cluster4, cluster5, and cluster16, 36.0%) [17, 18]. PBMC from patients showed a wide range of cluster types (Figure 3B). In comparison to controls, patients had a higher abundance of Neutrophils (cluster1), B cells (cluster10), T cells (cluster3, clusters4, cluster5, and cluster16), and mononuclear macrophages (cluster7, cluster12, and cluster15) (p<0.05, Figure 3C and D). By combining CD66b and CD11b, surface markers of granulocytes, with CD15 and CD16 expression on the subset of cluster1 (Figure 2C and D), we identified these cells as neutrophils [19]. Both cluster10 and cluster13 expressed classical surface markers of B lymphocyte cells (CD19 and CD20). Furthermore, CCR7 and CD80 molecules were expressed by cluster10 in patients, indicating that B lymphocytes from patients were in an activated state compared to the cluster13 in controls [20]. Among the three memory T cell subsets enriched in patients, they were divided into effector memory T cells (Tem, cluster4, and cluster5) and central memory T cells (Tcm, cluster16) based on CCR7 expression (Figure 2C and D) [21]. Additionally, cluster 16 and cluster4 expressed CD25, which could be two subsets of regulatory memory T cells (Treg) [22]. Based on the expression patterns of CD14 and CD16, cluster14 (CD14-CD16+) was considered a subset of non-classical monocytes (ncMo), while cluster15 (CD14+CD16+) was a group of intermediate monocytes (iMo) [23]. Cluster12 expressed CD1c, a molecule involved in lipid antigen processing, suggesting that it could be a population of dendritic cells [24], another subset of the monocyte-macrophage system. In contrast, some non-activated cells were the primary components of controls. CD4+ naïve T cells (cluster11), CCR7-CD80- B cells (cluster13), and ncMo (cluster14) were more enriched in controls (p<0.05, Fig. 3C and D).

We analyzed the functional molecules expressed in each of the immune cell clusters and found that patients exhibited higher levels of NF-kb in Neutrophil (Cluster1), CD4+CD8+ T cells (cluster3), CD8+ Tem cells (cluster4), and CD1c+ DC (cluster12), which suggested a proinflammatory activation state (Figure 3E) [25]. Moreover, macrophages (cluster7), CCR7+CD80+ B cells (cluster10), iMo (cluster15), and CD4+ Tcm cells (cluster16) from the PBMC of patients expressed higher levels of TNF-α than controls (Figure 3E). These findings suggest that the subsets enriched in patients exhibited a higher inflammatory activation state than those in controls.

### NKT, CD11b+CD4+ Tem, CD8+ TEMRA and CD163+ macrophages were the builders of the local immune environment.

**Figure 4.**
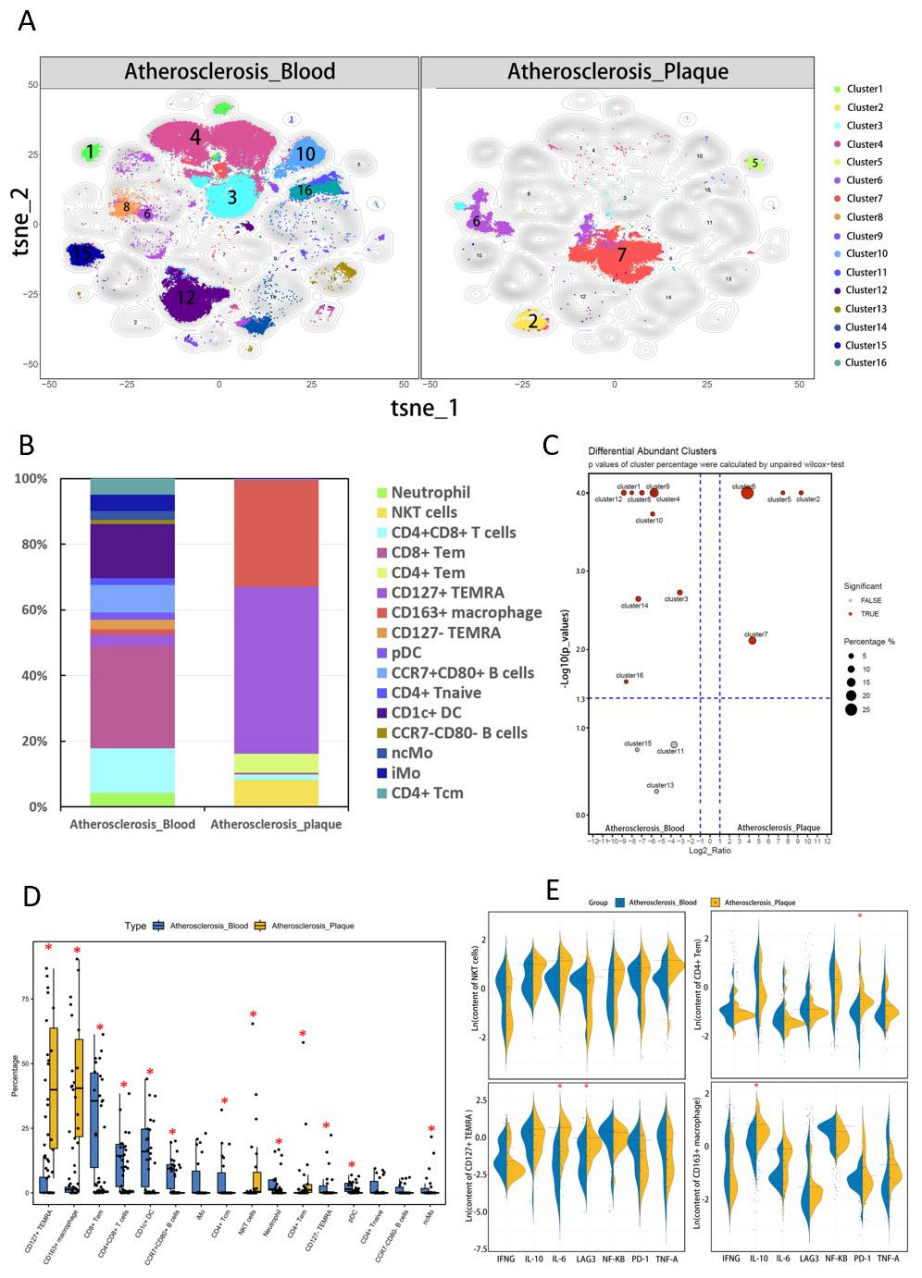
**Comparison of immune composition between atherosclerotic plaques (n=22) and paired blood (n=22)**. (**A**) The t-SNE dimension reduction method was used to display the components of plaques and paired blood. (**B**) A stacking histogram was used to show the proportion of each subset in plaques and blood. (**C**) The SPADEVizR method was used to analyze differences in immune composition between plaques and paired blood, and the results were presented on a volcanic map. (**D**) Boxplots were used to illustrate the difference in the percentage of each subset between plaques and paired blood (asterisks indicate subsets with statistically significant differences, P < 0.05). (**E**) (1-4): Beanplots were used to show the differences in functional status of subsets enriched in plaques compared to paired blood (asterisks indicate groups with statistically significant differences, P < 0.05).

To investigate differences in the immune environment, we compared the subset composition between atherosclerotic plaques and paired blood samples from 20 patients. Our results indicated that T cells and macrophages primarily constituted cells within the plaques. T cells could be further divided into three subsets (cluster2, cluster5, and cluster6). However, neutrophils (cluster1), monocytes (cluster14), dendritic cells (cluster9 and cluster12), and other T lymphocyte subsets (cluster3, cluster4, cluster8, and cluster16) were mainly enriched in paired blood (Figure 4C and D).

Cluster2, one of the three T cell subsets enriched in plaques, expressed both natural killer cell marker (CD56) and T cell marker (CD3), resembling a natural killer T (NKT) subset [26]. Our functional analysis indicated that cluster2 from plaques tended to secrete higher levels of TNF-α (p=0.061) and IL-6 (p=0.057) than their blood-derived counterpart, although the difference did not reach statistical significance (Figure 4E). Besides expressing markers of T memory cells, cluster5 also expressed CD11b on its surface (Figure 2C and D). This plaque-derived cluster exhibited higher PD-1 expression (p<0.05) than blood-derived ones (Figure 4E). CD45RA and CD45RO were co-expressed on the surface of cluster6 and cluster8. Notably, none of them expressed CCR7, suggesting that these cells may be two subsets of terminally differentiated effector memory cells (TEMRA) [27-29]. Cluster6, which was abundant in plaques, expressed CD127, while cluster8 enriched in peripheral blood did not. Our functional analysis revealed that plaque-derived cluster6 expressed a higher level of LAG3 (p=0.00034<0.05) and secreted more IL-6 (p=0.0029<0.05) (Figure 4E). CD8+ Tem (Cluster4) and CD4+ Tcm (cluster16) were the other two groups of memory cells mainly distributed in the peripheral blood of patients. Cluster7 was the most abundant macrophage subset in plaques. In addition to expressing the classic macrophage marker (CD68), it also expressed the haptoglobin receptor (CD163), which is a specific marker of M2 macrophages (Figure 2C and D) [30]. Furthermore, our functional analysis revealed that plaque-derived cluster7 produced more IL-10 than that from blood (p=0.0018<0.05), suggesting an anti-inflammatory role (Figure 4E).

**Figure 5.**
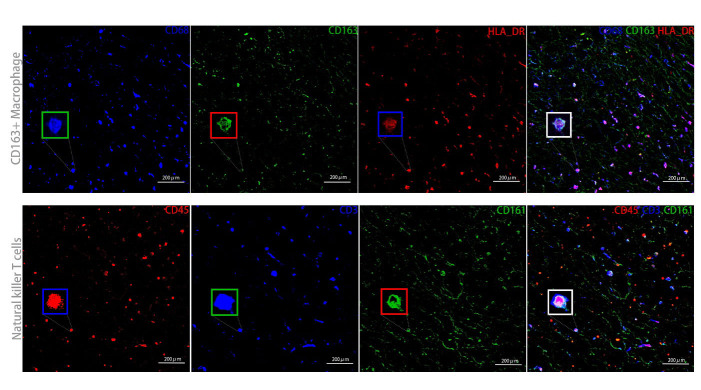
**Immunofluorescence staining of CD163+ macrophages and NKT cells in atherosclerotic plaques**. HLA_DR (red), CD163 (green), and CD68 (blue) was used to mark CD163+ macrophages (above) and CD161 (green), CD45 (red), and CD3 (blue) was used to mark NKT cells (bow).

To verify the identified cells in the plaque, we used HLA_DR, CD163, and CD68 to mark CD163+ macrophages, as well as CD161, CD45, and CD3 to mark NKT cells. Our results were consistent with the CyTOF data, indicating that CD163+ macrophages and NKT cells existed in plaques (Fig. 5).

### RNA-seq analysis of plaques and paired blood

**Figure 6.**
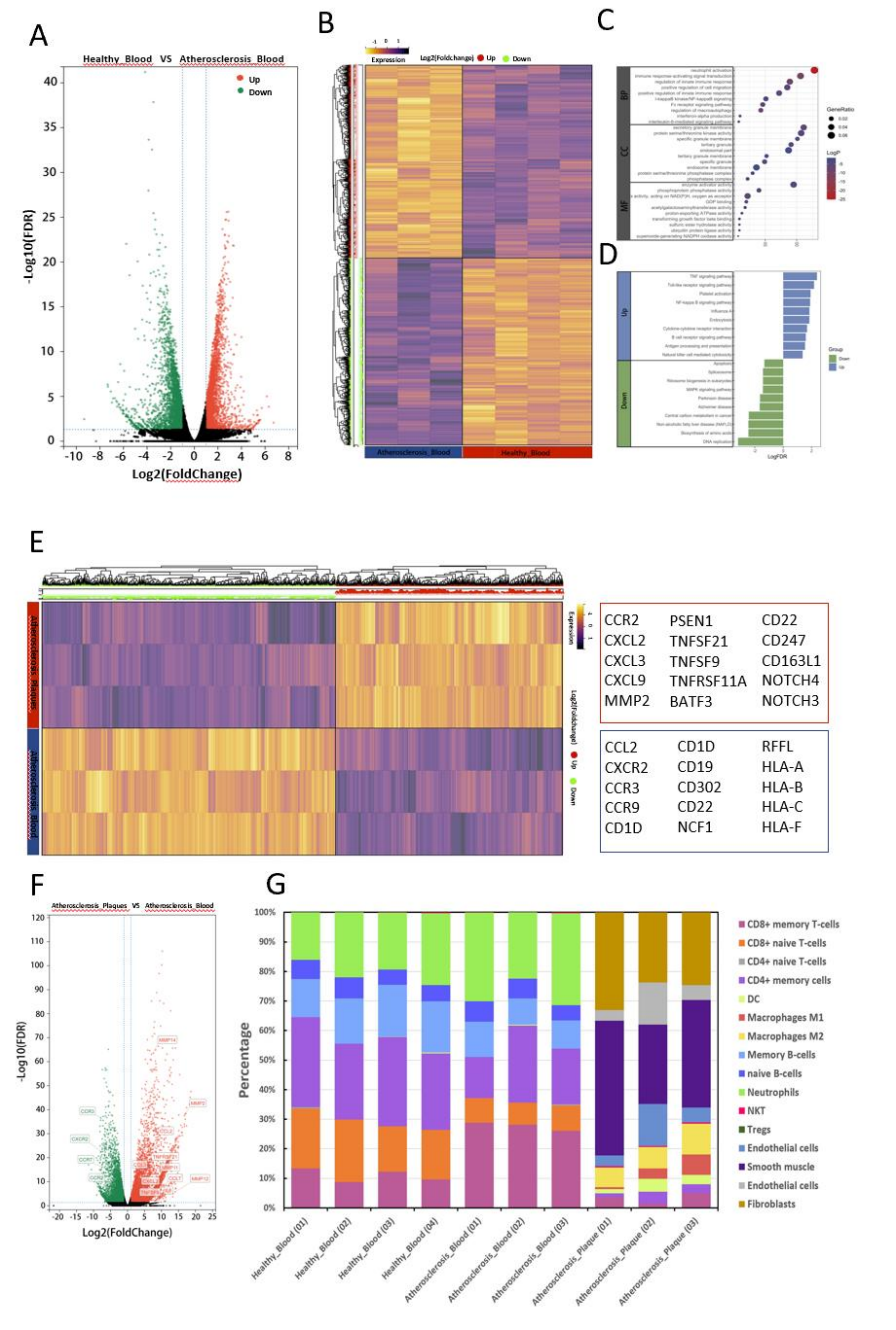
**RNA-seq analysis of peripheral blood from healthy controls (n=4), plaques (n=3) and paired blood (n=3) of atherosclerotic patients**. (**A**) Volcano plot showing differential gene expression between peripheral blood of atherosclerotic patients and healthy controls. (**B**) Heatmap displaying the expression pattern of differentially expressed genes in A. (**C**) Bubble map displaying the Gene Ontology (GO) results of up-regulated genes in peripheral blood of patients identified in A and B. (**D**) Histogram showing Kyoto Encyclopedia of Genes and Genomes (KEGG) pathway analysis results of up-regulated and down-regulated genes in blood of patients. (**E**) Volcano plot showing differential gene expression between plaques and paired blood of atherosclerotic patients. (**F**) Heatmap displaying the expression pattern of differentially expressed genes in E. (**G**) Stacking histogram showing the composition of immune cells in all samples inferred based on RNA-seq data using Xcell package.

To validate the findings from our previous CyTOF analysis, we performed RNA-seq analysis on peripheral blood samples from 3 patients and 4 controls. Our results indicated that 2770 genes were up-regulated in patients (defined as |log2FoldChange|>1 and FDR <0.05) (Figure 6A). To understand the functional implications of these up-regulated genes, we conducted pathway enrichment analyses. Our results showed that the NF-kappa B signaling pathway was significantly enriched in patients, according to both GO and KEGG analyses (Figure 6C and D), which was consistent with our CyTOF findings. Moreover, a large number of genes in patients were enriched in IL-6 signaling pathways by GO analysis and TNF signaling pathway by KEGG analysis (Figure 6C and D), which suggested an active inflammatory state in the peripheral blood of patients.

Subsequently, we conducted RNA-seq analysis on paired plaques and blood samples from 3 patients. Our results showed that 3786 genes were up-regulated in plaques (defined as |log2FoldChange|>1 and FDR <0.05) (Fig. 6E). Among these genes, several chemokine genes (CXCL and CCL) were up-regulated in plaques, while their receptor genes (CCR and CXCR) were up-regulated in paired blood (Figure 6E and F), indicating interactions between plaques and blood. The CD163L1 gene expressed by CD163+ macrophages was found to be up-regulated in plaques, which was consistent with the CyTOF analysis findings that cluster7 (CD163 macrophages) was the most abundant macrophage subset in the plaques. Additionally, we observed a higher level of PSEN1 in the plaques, which encoded a gamma-secretase component that cleaves Notch receptors expressed by T cells, leading to the activation and proliferation of T cells. We also found that other activated T cell genes (TNFSF21, TNFSF9, TNFRSF11A, BATF3) and genes involved in antigen presentation and T cell activation (CD22 and CD247) were up-regulated in plaques, which was also consistent with the results of CyTOF analysis.

Finally, based on these RNA-seq data, we utilized the xCell R package (Aran Dvir, 2017) to infer the proportion of each cell type in the samples. Our results indicated that peripheral blood samples mainly comprised of memory T lymphocytes (CD4+T and CD8+T cells), memory B lymphocytes, and neutrophils (Figure 6G), corroborating our CyTOF analysis findings. The immune environment of plaques mainly comprised macrophages (M1 and M2) and memory T lymphocytes (CD4+T memory cells and CD8+T memory cells) after excluding non-immune cells (smooth muscle cells, endothelial cells, and fibroblasts) (Fig. 6G), which was also consistent with our CyTOF analysis findings.

## DISCUSSION

Atherosclerosis is a chronic inflammatory disease characterized by lipid accumulation in the arterial wall [4]. The immune microenvironment within plaques is complex and consists of various immune cells. Furthermore, lesions located on the vessel wall are highly susceptible to changes in the circulatory environment [31, 32]. In this study, we used CyTOF to explore the disorder of the immune environment in both peripheral blood and plaques. We further validated our findings using RNA-seq and multicolor immunofluorescence. Our study comprehensively demonstrates the changes in the immune environment in patients from both an overall and local perspective.

Clinical studies have consistently shown that patients with systemic inflammatory diseases have a higher risk of atherosclerosis, including those with rheumatoid arthritis and systemic lupus erythematosus [33, 34]. Recent studies have also demonstrated the efficacy of systemic anti-inflammatory therapies, such as targeting IL-1β and trials with colchicine [35, 36]. These findings suggest that atherosclerosis is a systemic immune disorder characterized by local lesions. In this study, we used CyTOF to identify various immune cell subsets expressing activation markers in the PBMC of patients with atherosclerosis. We found that CCR7+CD8+B cells, enriched in patients, activated T lymphocytes using co-stimulatory molecule CD80 and have been shown to be pro-atherosclerotic in other studies [37]. We also observed that CD4+ Tem expressing CD11b were more aggregated in the PBMC of patients compared to controls, and these cells showed significant frequency changes between plaques and paired blood. The iMo subset, which is functionally linked with neo-vascularization in advanced plaques, was more abundant in the PBMC of patients [38]. Moreover, we found that subsets enriched in PBMC of patients expressed higher levels of NF-kb and TNF-a, which are broadly involved in the inflammatory response [39]. These findings were also validated by RNA-seq analysis, which showed that the genes of chemokines and their receptors were respectively concentrated in PBMC and plaques, indicating an interaction between circulating and lesioned cells. These changes in the proportion of different functional subsets from the same cell type cannot be found based on the method of rough cell classification. Patients with atherosclerosis and normal blood routine may be in a potential "sub-inflammatory state," which may also account for the effectiveness of systemic anti-inflammatory therapy. Our study comprehensively demonstrated the changes of immune environment in patients from both the overall and local perspective using CyTOF, RNA-seq, and multicolor immunofluorescence.

Macrophages are known to play a critical role in the growth and rupture of atherosclerotic lesions [40]. However, recent studies have highlighted the heterogeneity of macrophages and their ability to change phenotype expression in response to environmental cues [41]. In this study, a cluster of CD163+ macrophages was found mainly in plaques, indicating an anti-inflammatory shift within the lesion. CD163, a haptoglobin receptor, promotes macrophages to clear inflammatory substances and is typically distributed on the surface of M2-like macrophages [42, 43]. Interestingly, CD64 and CD11c, known as M1 macrophage surface markers, were also expressed on the surface of these CD163+ macrophages, suggesting a transitional state of this subset [44]. Therefore, strategies to switch macrophage phenotypes may help in plaque regression. T cells have also been identified as important contributors to the local immune microenvironment [10, 45, 46]. In the comparison between plaque and paired blood, three subsets of T lymphocytes were found to be concentrated in plaques, two of which expressed memory markers, and the other was NKT cells. NKT cells are a bridge between the innate and adaptive immune systems and play a role in the recognition of lipid antigens [47, 48, 49]. In this study, NKT cells in plaques secreted higher levels of pro-inflammatory cytokines, IL-6 and TNF-a, than those from blood, suggesting their pro-inflammatory polarization involved in the construction of the local immune environment. Memory cells, on the other hand, offer long-term protection against pathogens and play a critical role in chronic inflammation [50]. However, repetitive exposure to antigens may induce the accumulation of exhausted memory T cells [51]. In this study, CD11b+ Tems expressed higher levels of PD-1 and CD127+ TEMRA expressed higher levels of LAG3 compared to their counterparts in blood. Inflammatory activation of these memory cells within the plaques was also observed. These memory cells, characterized by high cytotoxicity, low proliferation, and sensitivity to apoptosis, have been associated with excess inflammation and age-related chronic inflammatory diseases [51]. Specific therapies targeting these memory cells within plaques may contribute to the regression of atherosclerotic disease.

In conclusion, our study provides an atlas of the immune landscape in atherosclerotic lesions. We found that pro-inflammatory activation is a major characteristic of peripheral blood in patients with atherosclerosis. NKT cells, CD11b+ CD4+ Tem cells, CD8+ TEMRA cells, and CD163+ macrophages are all key players in the local immune environment. Among these, NKT cells, CD11b+ CD4+ Tem cells, and CD8+ TEMRA cells, which exhibit pro-inflammatory activity, may be associated with lesion progression. The precise immune profile of atherosclerosis and the subsets identified in the comparative analysis will help us further understand the complex pathophysiological mechanisms of atherosclerosis and provide direction for clinical-specific anti-inflammatory treatments.

### Limitation

Firstly, the uneven distribution of hyperlipidemia between patients and controls may have influenced the results of the circulating immune environment analysis. Secondly, the higher use of statins and antihypertensive drugs in patients could also have affected the immune environment. Additionally, some antibodies that could distinguish certain immune cell subsets were not utilized, which may have resulted in imprecise classification. Lastly, it should be noted that patients undergoing carotid atherectomy typically have advanced carotid stenosis and surgically treated atherosclerosis, meaning that the results of the local immune microenvironment analysis may not be representative of the immune characteristics of early atherosclerosis. Therefore, the local immune environment analysis presented in this study may not accurately reflect the immune characteristics of early-stage atherosclerosis.

## Supplementary Materials

The Supplementary data can be found online at: www.aginganddisease.org/EN/10.14336/AD.2023.0426-1.


