# Supplementary material for: Single-Cell Atlas of Atherosclerosis Patients by Cytof: Circulatory and Local Immune Disorders
Source: Aging Dis. 2024 Feb 1;15(1):245–58. doi: 10.14336/AD.2023.0426-1 (PMC10796097; doi:10.14336/AD.2023.0426-1)
Supplement: Supplementary file 1 [file AD-15-1-245-s.pdf]

SUPPLEMENTARY DATA

Single-Cell Atlas of Atherosclerosis Patients by Cytof: Circulatory and Local Immune Disorders

Xiaolong Ya, Hao Li, Peicong Ge, Yiqiao Xu, Zechen Liu, Zhiyao Zheng, Siqi Mou, Chenglong Liu, Yan Zhang, Rong Wang, Qian Zhang, Xun Ye, Wenjing Wang, Dong Zhang, Jizong Zhao

© 2023. Ya X et al. Published online at http://www.aginganddisease.org/EN/10.14336/AD.2023.0426-1

SUPPLEMENTARY DATA

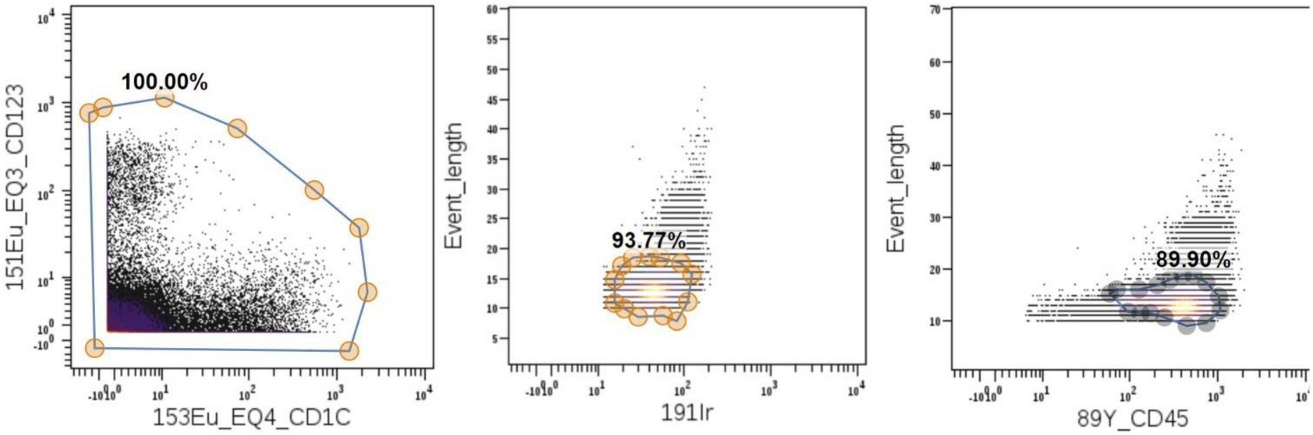

Supplemental Figure 1. Date cleaning in Cytobank. Breads were removed from the data using EQ4 and EQ3 channels. Subsequently, single cells were selected using Ir channels, and finally CD45+ cells were selected for downstream analysis using CD45 channels

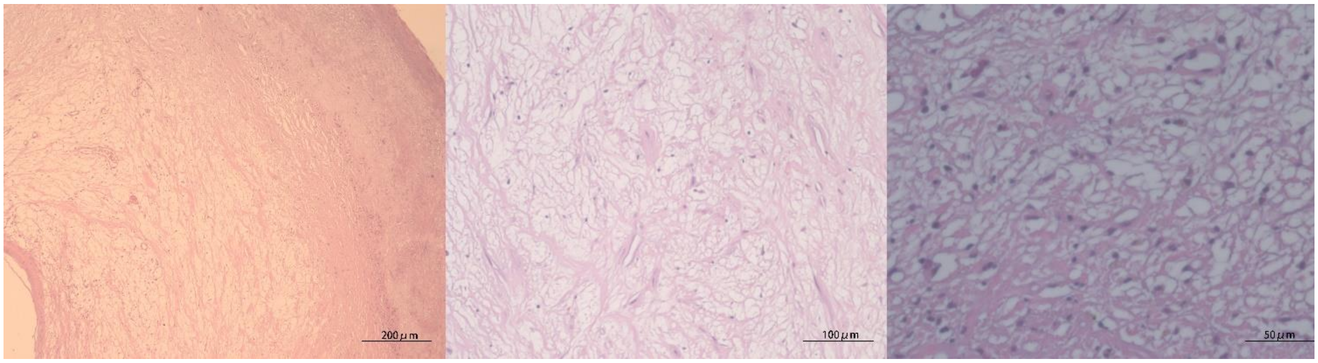

Supplemental Figure 2. H&E staining of atherosclerosis plaque tissue. Before immunofluorescence, the sections were stained with HE to confirm the quality and integrity. In addition, the tissue structure was identified.

Supplemental Table 1. Demographic and Clinical Features of the CyTOF Cohorts

Parameters	Controls (n=20)	Patients (n=22)	P-value
**Basic characteristics**			
Gender (male/female)	17/3	18/4	0.782
Age (year, Mean±SD)	64.58±5.13	65.09±5.05	0.747
BMI (kg/m2)	19.04±3.74	19.86±4.13	0.505
**History**			
Drinking, n(%)	11(55.0%)	13(59.1%)	0.789
Ever smoker, n(%)	14(70.0%)	17(77.3%)	0.592
Hypertension, n(%)	9(45.0%)	15(68.2%)	0.130
Dyslipidemia, n(%)	6(30.0%)	17(77.3%)	0.002
Diabetes, n(%)	4(20.0%)	7(31.8%)	0.379
**Medications**			
Statins, n (%)	3(15.0%)	19(86.4%)	<0.001

© 2023. Ya X et al. Published online at http://www.aginganddisease.org/EN/10.14336/AD.2023.0426-1

Aspirin, n (%)	3(15.0%)	16(72.7%)	<0.001
Antihypertensive, n (%)	5(25.0%)	12(54.5%)	0.051
Antidiabetics, n(%)	4(20.0%)	6(27.3%)	0.305

Supplemental Table 2. Mass cytometry antibodies panel design.

Antigen	Symbol and Mass	Antibody clone	Source
CD45	89	HI30	Fluidigm
CD3	111	CD3-12	Abcam
CD4	116	EPR6855	Abcam
CXCR3	141	49801	R&D
CD19	142	HIB19	Fluidigm
CCR7	143	15503	R&D
cd31	144	WM59	Fluidigm
CD16	145	3G8	Fluidigm
CD45RA	146	4KB5	Abcam
CD86	147	37301	R&D
NFKBP65	148	E379	Fluidigm
cd194	149	L291H4	Fluidigm
CD11C	150	ICRF 3.9	R&D
CD123	151	6H6	Fluidigm
TNF-a	152	Mab11	Fluidigm
CD1C	153	EPR23189-196	Abcam
CD25	154	YTH906.9HL	Abcam
PD-1	155	EH12.2H7	Fluidigm
CD14	156	HCD14	Fluidigm
CD33	158	WM53	Fluidigm
CD15	159	SP159	Abcam
CD64	160	AT17-2	Abcam
CD66B	161	EPR25354-2	Abcam
CD8	162	RPA-T8	Fluidigm
IL-10	163	EPR114	Abcam
CD56	164	EPR21827	Abcam
CD127	165	EPR23747-333	Abcam
CD45RO	166	T200/197	Abcam
CD20	167	Hu2	R&D
CD206	168	15-2	Fluidigm
CD24	169	ML5	Fluidigm
IL-6	170	EPR21711	Abcam
CD80	171	16-10A1	Fluidigm
CD163	172	EPR19518	Abcam
CD68	173	298807	R&D
HLA-DR	174	L243	Fluidigm
LAG3	175	11C3C65	Fluidigm
IFNg	176	EPR22171	Abcam
CD11B	209	EP1345Y	Abcam

© 2023. Ya X et al. Published online at http://www.aginganddisease.org/EN/10.14336/AD.2023.0426-1
